# Supplementary material for: Alteration of Pituitary Tumor Transforming Gene 1 by MicroRNA-186 and 655 Regulates Invasion Ability of Human Oral Squamous Cell Carcinoma
Source: Int J Mol Sci. 2021 Jan 20;22(3):1021. doi: 10.3390/ijms22031021 (PMC7864193; doi:10.3390/ijms22031021)
Supplement: Supplementary file 1 [file ijms-22-01021-s001.pdf]

Supplementary Table

Table S1. Antibody lists used in the study

Antibody	Sources	Dilution factor	Catalog number	Company
PTTG1	Rabbit	1 : 1000	34-1500	Invitrogen
p-mTOR(Ser2448)	Rabbit	1 : 1000	5536	Cell signaling
mTOR	Rabbit	1 : 1000	2983	Cell signaling
p-AKT(Ser473)	Rabbit	1 : 1000	9271	Cell signaling
AKT1	Mouse	1 : 1000	Sc-5298	Santa Cruz
P53	Rabbit	1 : 1000	LF-PA0050	Ab frontier
Cycline E	Rabbit	1 : 1000	630702	Bio Legend
gp130	Rabbit	1 : 1000	Sc-656	Santa Cruz
p-STAT3(Ser727)	Rabbit	1 : 1000	9134,9131	Cell signaling
STAT3	Rabbit	1 : 1000	Sc-483	Santa Cruz
p-Erk(Thr202/Tyr204)	Mouse	1 : 1000	9106	Cell signaling
Bcl-2	Rabbit	1 : 1000	Sc-492	Santa Cruz
Bak	Rabbit	1 : 1000	1542-1	Epitomics
Cytokeratin peptide18	Mouse	1 : 500	C8541	SIGMA
Vimentin	Goat	1 : 500	V4630	SIGMA
p-FAK(Tyr397)	Rabbit	1 : 1000	3283	Cell signaling
Integrin alpha4	Rabbit	1 : 1000	4827	ProSci
Integrin alpha5	Rabbit	1 : 1000	610634	BD biosciences
Integrin beta1	Rabbit	1 : 1000	MAB1778	R&D systems
Integrin beta7	Mouse	1 : 500	MAB4669	R&D systems
Rho A	Rabbit	1 : 1000	2117	Cell signaling
ROCK1	Rabbit	1 : 1000	4035	Cell signaling
GAPDH	Rabbit	1 : 3000	LF-PA0018	Abfrontier
